# The Value Chain Approach in One Health: Conceptual Framing and Focus on Present Applications and Challenges

**DOI:** 10.3389/fvets.2017.00206

**Published:** 2017-12-11

**Authors:** Nicolas Antoine-Moussiaux, Marisa Peyre, Pascal Bonnet, Charles Bebay, Mohammed Bengoumi, Astrid Tripodi

**Affiliations:** ^1^Fundamental and Applied Research for Animals and Health (FARAH), University of Liège, Liège, Belgium; ^2^Agricultural Research Centre for International Development (CIRAD), Montpellier, France; ^3^FAO ECTAD West and Central Africa, Accra, Ghana; ^4^FAO Subregional Office for North Africa, Tunis, Tunisia; ^5^FAO, Animal Health Service, Rome, Italy

**Keywords:** livestock value chain, behavioral analysis, socioeconomic, One Health, interdisciplinarity

## Abstract

The value chain (VC) is a major operational concept for socioeconomic analysis at meso level. Widely mobilized in development practice, it is still undergoing conceptual and practical refining, e.g., to take account of environmental and social sustainability. Briefly, VC refers to a system of value creation through the full set of actors, links, technical and commercial activities and flows involved in the provision of a good or service on a market. In the past decade, this concept has been promoted in the management of animal health. In particular, the emergence of highly pathogenic avian influenza (HPAI) has triggered an interdisciplinary dynamic including VC analysis as a central tool. These efforts promoted participatory investigation methods in the analysis of health systems. Using qualitative and quantitative data, these methods acknowledge the usefulness of actors’ involvement and knowledge, hence facilitating the transdisciplinarity needed for effective action. They fit into adaptive and action-oriented strategies, fostering stakeholders’ participation. Recent research on HPAI surveillance in South-East Asia merged VC and participatory approaches to develop innovative tools for analyzing constraints to information flow. On-going interventions for HPAI prevention and control as well as the prevention of other emerging zoonotic risks in Africa are presently building on this VC framework to develop strategies for its application at national and regional scales. Based on the latter experiences, this article proposes a field-based perspective on VC applications to animal and public health systems, within a One Health approach responding to the overall challenge of complexity.

## A Complexity Framework of Animal and Public Health

Animal and public health issues are increasingly considered as “complex,” referring to challenges as emerging zoonoses, antimicrobial resistance or environmental contamination, while ensuring food security for a growing population. These so-called “wicked problems” are strongly interconnected, tied to the rapid evolution of animal production, trade, as well as intricate and widely unpredictable epidemiological processes involving shared pathogens inside a shared and changing environment. Considering the case of avian influenza, the wide diversity of actors across food systems at different country scales is source to diverse and even conflicting interests impacting the issue; the unpredictable virus mutations or reassortments, the involvement of wild birds in the virus spread, the unregulated poultry production and trade in many countries, and the potential for virus spillover to other species make surveillance paramount in humans, domestic animals and wildlife, in a context of weak cross-sectorial collaboration. Recognizing this “complexity” is an important step, pointing to a conceptual framework guiding practical action (1). Fitting into the theory of systems (2), complexity results from the large number of components in open systems, interwoven by non-linear and feedback interactions, as observed in epidemiological and economic relationships inside food systems. Due to emergent properties of systems, complexity calls for holistic approaches in health management, translated into transdisciplinarity, joining academic and non-academic knowledge and action to solve societal problems (3). The openness of complex systems may be exemplified by animal production intensification within poorly regulated framework: this may point to defaults in biosecurity, causing higher risk for geographic spread and zoonotic transmission. But it also refers to the uncontrolled use of antibiotics, to food contamination and environmental damage, etc. This openness means that any intention to analyze such systems in view of addressing health problems will first need a cautious framing of their boundaries, defining the limits and outreach of the proposed solutions.

This complexity framework is structuring a range of approaches in animal and public health pointing to the communality of health issues and the need for an integrated management. The most prominent concepts are probably the EcoHealth or the One Health (OH) approaches (3). To analyze health problems within the entanglement of their drivers and consequences, systems have to jointly represent interactions between humans, animals, and their environment, leading to the notion of social–ecological system and the structured analysis of their subsystems along a diversity of methods (4). Such an understanding needs a tight collaboration of multiple disciplines, among which social sciences hold a crucial role, since human behavior and governance of systems’ components are central drivers of the modeled challenges (5, 6).

## Socioeconomic Reasoning for OH

The common misunderstanding of economics as being a “science counting in monetary terms” instead of “studying behavior” has restricted its contribution to a role of accountancy of disease impact and management (7, 8). Presently, the OH concept, highlighting complexity and the deficits of mono-dimensional approaches, reactivates the already identified need to question economic methods and frameworks (9). Deciphering whether certain forms of economic organization, coordination, or behavior are particularly prone to higher health risk appears now as a major scientific challenge.

This article presents the value chain (VC) approach as a typical example of socioeconomic reasoning. A particular feature of it lies in its attempt to address multiscale systems, applying its conceptual framework and tools to individuals, households, firms, communities, networks, countries, and at the international level. It considers both the influence of individuals on the group and the influence of the group on the individual, this cross-determination being a textbook application of feedback interactions inside complexity frameworks (10). As a matter of fact, VCs are complex in nature. Indeed, beyond the simplified models proposed by economists to tackle wide economic sectors, empirical research on livestock VCs shows the coexistence of interwoven subsystems encompassing various networks of stakeholders, forms of coordination and levels of complexity: informal or formal, modern or traditional, high or low technology, quality-, or price-driven chains (11).

## Value Chains and OH

Value chain is a major operational concept in business and economic literature, built upon the seminal works of Porter (12). Widely implemented in agricultural development, VCs are subject to various approaches and practical guides (13). In its widest understanding, VC refers to the full set of actors, their mode of interaction (types of agreements, relations) within strategic networks, activities (technical or economic functions), and flows (material, immaterial) involved in the provision of a good or service on a market. May also be included in the same approach peripheral actors, not taking directly part to the product or service provision, but supporting or influencing VC behaviors and strategies (14). Hence, the components of VC analysis are: mapping actors and processes, understanding governance, identifying opportunities for upgrading and improving equity (15). The overall upgrade of VC may call for changes in the legal framework and for financial or technical support, to accompany individuals in improving their process in the VC (upgrading), recentering it on a core-business (downgrading), or redirecting their activities as they are unable to cope with changes in the VC (out-grading) (16).

Considering both actors and processes inside a governance structure, VC analysis appears as a typical case of socioeconomic reasoning. Governance is here a central concept gathering all forms of coordination between actors: vertical and horizontal, internal and external. It covers the diversity of rules and frameworks influencing actors’ behavior (regulatory structure, private standards, cultural norms, contracts), which all may constitute incentives for practices entailing animal and public health risks or benefits. Each actor, by contributing to the process, is contributing part of the final product/service value, termed “value added.” This value creation is central to economic analysis, aiming at increasing and sharing it among stakeholders. However, the understanding and fostering of change in VC governance may pursue diverse goals, such as food safety, animal welfare, and public health, thus fitting in the OH framework (17). Recently, FAO renewed the approach proposing the concept of sustainable food VC (SFVC), extending the coverage to social and environmental dimensions of sustainability by taking account of externalities in the calculation of value added for society (18). Hence, beside any particular health focus, VC improvement increasingly calls itself for interdisciplinary research (19), e.g., to better include smallholders in sectorial growth.

The OH perspective mainly addresses animal-related VCs, i.e., livestock VC themselves and related chains of feed and health service provision, but also wildlife and bushmeat VC. These are contributing to food security, and thus to health, but are also a source of risks that have to be controlled along the chains of production, technical processes and stakeholder relations. The main risks considered are zoonoses, foodborne toxicoinfections, drug residues, and other chemical contamination. The threats to environment and their consequences for human health are also relevant problems to be addressed through livestock VCs, as highlighted in the SFVC approach. Technical approaches have long been developed for risk control through procedures under normative quality standard frameworks, as the Hazard Analysis and Critical Control Point, but these are mostly restricted to the industrial context. As exposed here below, VC approach in a OH perspective goes beyond that sole technical control to encompass a wider notion of actors’ behavior and explicitly address the link to risk dynamics outside the strict processing of products.

In the past decade, the emergence of highly pathogenic avian influenza (HPAI) acted as a triggering event in the building of the OH approach, gathering first public and animal health actors in a common management of the crisis (20). These interdisciplinary efforts mobilized VC analysis as a tool to map actors, processes and value creation in order to plan HPAI control and assess the impact of the disease and control measures (21–23). In HPAI management, as in animal health in general, VC analysis was firstly used for impact assessment of diseases and/or interventions. The potential of this framework for behavioral studies in risk management and health governance appeared later (15, 24). Mainly drawing on the experiences of HPAI and Rift Valley Fever, the importance of understanding VC actors’ behaviors and strategies contributing to risk production and management was progressively affirmed. Hence, further research was called for and developed, again in the framework of HPAI. Recurrently, published and unpublished works highlighted both the importance of VC analysis for health governance and safety management in the livestock sector, but also the limits of it (e.g., lack of social and spatial perspectives in most VC research) and the need for methods to evolve and adapt to the specific needs of this OH application (24–26). More precisely, participatory approaches and qualitative research have proved crucial in view of needed changes.

## VCs and Participatory Approaches

Value chain analysis for HPAI control was embedded in a movement toward the use of participatory investigation methods (21, 23), thus reviving participatory epidemiology (27) and the consideration for farmers’ viewpoint (28). These methods, using qualitative and quantitative data, acknowledge the usefulness of field actors’ knowledge and spur actors’ involvement, hence facilitating the transdisciplinarity needed for analysis and action. The wide use of visual tools in participatory approaches facilitates communication, information sharing, and joint decision-making, with an explicit goal of triggering positive changes in communities. This philosophy of action-oriented intelligence also underwent important developments in parallel in animal and public health (29, 30). Within the OH approach, community participation, supported by shared policy between environment, human and animal health, proved its efficacy in the surveillance of vector-borne diseases and zoonoses (31), while the eradication of rinderpest represented a major contribution to world food security (29).

Originating in action-research, participatory approaches emerged as a good practice in VC analysis for risk management purposes. There are two main motives for this wide adoption. First, VCs are highly variable in length, complexity, and degree of formalized organization, thus calling for flexible and non-standardized methods. The particular weight of home-consumption and direct sale to consumer is part of this diversity of practices. Also, in developing and transition economies more particularly, much of the economic activity pertains to the informal sector, hence partly hidden, though underpinning very concrete networks in action. Therefore, information is poorly accessible by formal surveys gathering accountancy data with quantitative goals, as performed in classical VC analysis, and actors may not be easily identified and mobilized for actions. Second, the goal is here to derive a thorough understanding of behaviors, motives, and strategies that are relevant to both food chains and health risk management, in order to envision systems’ evolution and the conditions for positive change. Therefore, qualitative information on behaviors and motives are paramount, again calling for a step away from classical VC analysis.

Finally, participatory approaches brought the flexibility to combine the structural aspect of VC analysis with behavioral information, given the diversity of bonds between actors, the variety of agents’ types, as well as the diversity of motives and risks.

## VCs and Behavioral Analysis: Method and Highlights

In the continuity of the sustained effort for HPAI control in South-East Asia, participatory VC approaches were used as innovative tools to analyze constraints to HPAI surveillance (32, 33). These studies focus on concepts of incentives and governance, inside a flexible, participatory approach along the poultry VCs, to understand the information flow about animal mortality cases in Vietnam and Thailand. Methods used are mapping of stakeholders, describing actors and relationships between them and further investigations through in-depth qualitative interviews, social network analysis and stated preference methods. These approaches, qualitative in nature, are complementary to another thread of research in the field, rather quantitative, that also mobilizes VCs to understand and prevent HPAI risk in Vietnam from the starting point of markets (34). Joining such systematic and quantitative market characterizations to a wider and more in-depth analysis of VC appears as the backbone structuring the present approach in the context of the prevention of emerging pandemic threats and in the emergency control of HPAI in different countries. Other quantitative modeling approaches applied to VC analysis present potential to contribute to OH-oriented VC analyses, such as system dynamic modeling (35–37). These indeed fit in the here-proposed framework of complexity, taking into account feedback loops, and may implement participatory approaches in building of the models (37). Presently focusing on economic output, sometimes based on health management choices (36), dynamic modeling may account for diverse risky practices and be bound to epidemiological models to generate health-related outputs and perform in-depth risk analysis.

**Figure 1:**
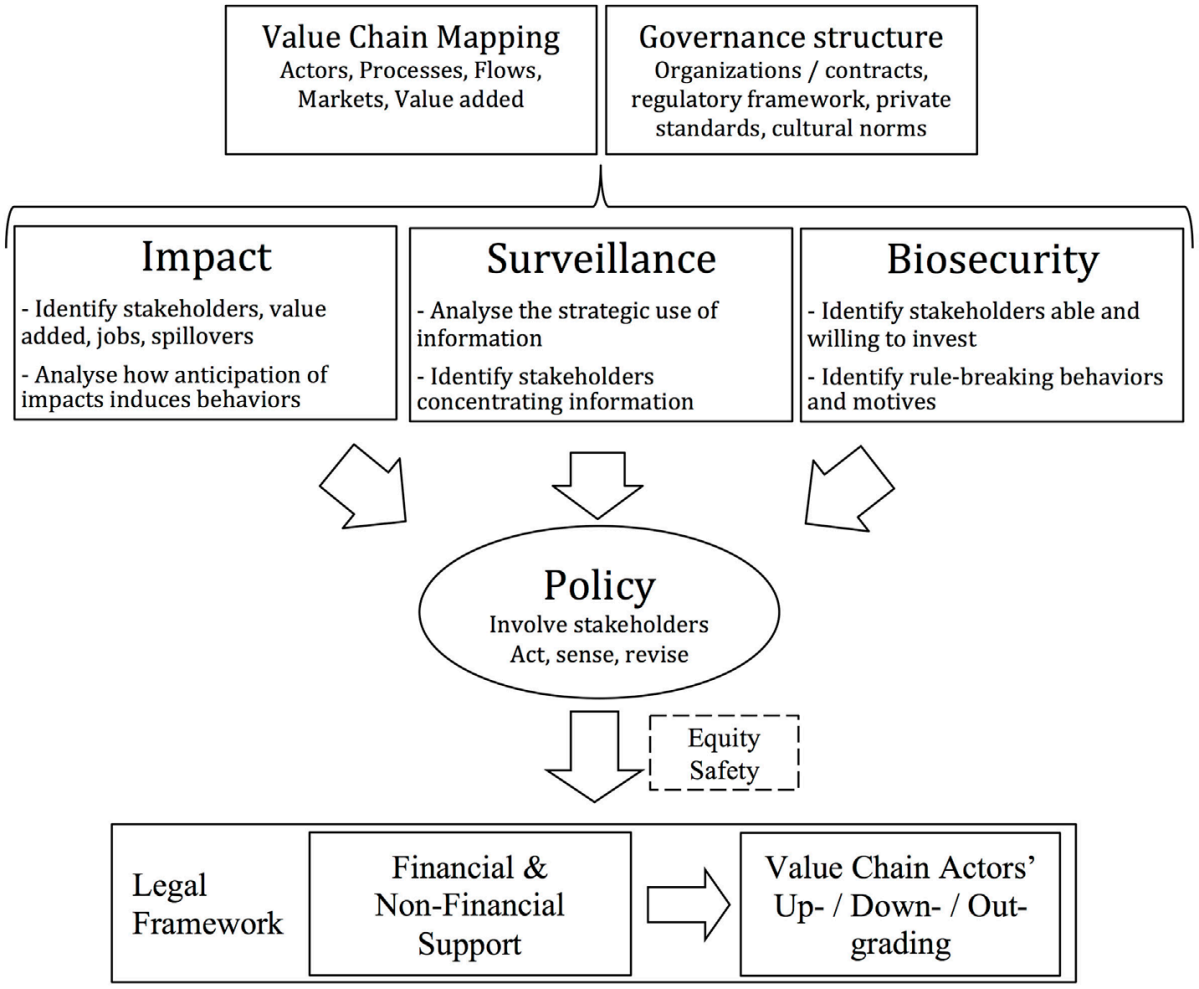
Main threads in value chain analysis and upgrading for health management purposes.

Livestock VC analysis for health risks management may be framed around three main themes: impact, surveillance, and biosecurity (Figure 1). To illustrate the approach, practical aspects, and behavioral highlights of VC analysis for health risk management are described here.

Regarding impact analysis, VCs are taken in their primary role of understanding value generation and distribution among stakeholders, considering their diversity and numbers at each level. In current applications, impacts on jobs hold a particular importance in the political agenda for HPAI management in Maghreb, hence influencing public strategies under planning. Within a behavioral perspective, impact analysis presents two main contributions. First, health crisis management results in structural effects on VCs, as in Vietnam where HPAI and its public management reinforced the large intensive poultry production, against the interests of smallholders (38, 39). Modifying power and competition relationships, these structural effects lead to feedback loops between the VC structure and the health risk. Second, actors’ anticipations of these impacts drive their decision-making, influencing the risk itself and its impact, e.g., poultry producers anticipate impacts of epizootics on prices and then develop speculative strategies intending to engage in contra-cyclical stocking and selling (33). These anticipations are imperfectly informed and subject to perceptual bias. However, since our interest lies in actors’ behavior, relevant data to gather indeed consist in perceptions rather than factual impacts only. Other examples of anticipations by VC stakeholders may concern control measures, market, and farm-gate prices due to mortality, panic, or bargaining effects (33, 40). How impacts are anticipated and affect decision-making is thus crucial in understanding VCs’ reaction facing disease risks.

The latter elements are illustrative of the strategic role of epidemiological information inside VCs. Indeed, while public and animal health surveillance may be considered as public goods, even global public goods, these may be rather managed as private or collective ones by VCs actors. Considering the continuum between data, information, intelligence, and knowledge, we observe that information in VCs is often used as strategic intelligence for private use, thus influencing behavior. Within VC analysis, the value produced by each actor and the weight of these actors at each VC level is indicative of stakes in this strategic intelligence, thus incentives to gather disease information and hide or disclose it to targeted business partners. Also, the scale of operation of an actor will determine the geographic area covered by such a private surveillance, defining the so-called epidemiological territories of VC actors (41). This scale of operation and concentration at one level of VC will also inform about actors’ relative power and ability to influence others’ decision-making. Mutual behavioral influence will typically result from vertical integration along the VC, i.e., contracts or ownership bonds between different VC levels, or other commercial links, e.g., credit/debt and persistent network relationships. In the prospect of a participatory management of health risks, these important players identified through VC analysis will be considered as main partners or on the contrary main offenders in the joint management of health information as a public good. Whatever the situation, a first step is to understand the strategic positioning of each actor. Hence, understanding how VC governance affects health information use and epidemiological intelligence should be central to any VC analysis for health management.

The third framing theme is biosecurity, considered in a wide understanding, as being all actions that may be undertaken individually or collectively to prevent the risk studied. A main objective of VC analysis in this respect is to identify the stakeholders able and willing to invest and presenting objective interests in adopting or leading the needed changes. Both aspects relate to the value produced at the actor’s level and its access to financial services (insurance and credit), which are central to VC analysis and possible public action to support VC. Again, governance is here crucial due to the externalities of biosecurity measures and the classical coordination problems in managing commons (42). Indeed, besides a vision centered on formal VC actors being partners of its improvement, biosecurity along VC crucially depends on the development of informal and rule-breaking practices. Such practices may result from a lack of access of those actors to the formal VC, due to financial, regulatory, organizational, technological, or even cultural barriers. In a wider understanding, these may be typical free-rider behaviors, motivated by private benefits of not respecting standards and regulations in a context of weak rules enforcement. A difficult but needed judgment to be conducted by regulatory bodies is to understand which actors have to be included in the reinforced VC and which should be controlled to protect other VC actors from their detrimental behaviors. Hence, understanding behaviors and attitudes of VC stakeholders toward biosecurity and identifying incentives or barriers to adhere to biosecurity is crucial for adapting control measures and is part of the participatory analysis of VC.

## Opportunities, Limits, and Challenges of VC Analysis

The overall understanding of health challenges within social–ecological systems should benefit from wider and deeper VC analysis. Fundamentally, VC represents a fruitful conceptual framework to analyze actors’ behavior and strategies at different levels of the complex systems under consideration. It helps framing complex health problems, guiding action during investigation and triggering change. Although methods envisioned are making several steps away from classical VC analysis to adapt to health complexity, methodological challenges remain, being under constant revision throughout their current application. As detailed below, these challenges come down to a question of framing or setting the problem’s boundary, in terms of diversity of actors and processes (including VC supporters and influencers, related VCs), in terms of scales (local, regional, or international markets), or in terms of dynamic (single capture vs. follow-up). The aforementioned question of application of VC analysis to bushmeat and wildlife-related zoonoses is also a topic of current methodological challenges.

The frameworks, as here proposed and presently implemented, present a weakness in not fully taking environmental aspects into account. The SFVC approach and the Life Cycle Analysis methods (43) might be built upon to develop a consistent integrated approach. A more intrinsic limit of VC approach is its focus on a single product and the inclusion of actors in their sole link to this product, with little interest to study inter VC interactions, VC impact on ecosystems, or impact of all VC on inclusive local development. This is particularly constraining in developing countries, where livestock have multiple contributions to livelihoods with known implications in health and environmental questions (44). Also, livestock-keeping households are mostly running multiple activities, as a risk management strategy. Due to this function as well as to epidemiological consequence for cross-species pathogen transmission or joint risk production, this importance of diversification is not to overlook. An additional limit of VC analysis, also particularly constraining in rapidly changing contexts of developing and transition countries, is that it provides a relatively static image of the meso-system studied. Again, a methodological renewal, based on rapid assessment approaches and targeted continuous data collection could help overcoming this issue. However, while tackling all these limits inside the VC framework might appear as interesting methodological challenges, a more direct consequence is the need to keep an awareness of these and more directly overcome them where deemed important by joining other disciplines and frameworks in a behavioral analysis.

## Conclusion

This article presents how VC analysis contributes to behavioral understanding and change in OH issues. It defends the value of participatory and qualitative approaches, which in turn present an important limit to standardized, multiple countries projects as presently developed for the management of zoonoses and emerging pandemic threats. Obviously, this scaling-up question will remain a matter of trade-off between extensiveness and depth of investigation, as basically taught in social sciences classes. While the needed participatory process is flexible and qualitative in nature, therefore weakly standardized, the joint use of more systematic and quantitative approaches, as markets and flows characterization, risk analysis and system dynamics, is an important answer to generate consistent data to guide decision-making at the national and international levels. This, finally, is not to overlook the basic philosophy of participatory approaches that lies in the fostering of bottom-up changes. Finally, tackling complexity will remain a matter of trade-offs, flexibility, and multiple perspectives.

## Author Contributions

NA-M: joint experience analysis, writing: drafting, inputs coordination, and finalization. MP: joint experience analysis as CIRAD project leader, and cowriting. PB: joint experience analysis as CIRAD project leader and cowriting. CB: joint experience analysis as FAO-ECTAD West and Central Africa project leader and cowriting. MB: joint experience analysis as FAO-Maghreb project leader and cowriting. AT: joint experience analysis as FAO Animal Health Service officer and co-writing.

## Conflict of Interest Statement

The authors declare that the research was conducted in the absence of any commercial or financial relationships that could be construed as a potential conflict of interest.
